# Uniportal and three-portal video-assisted thoracic surgery pulmonary lobectomy for early-stage lung cancer (UNIT trial): study protocol of a single-center randomized trial

**DOI:** 10.1186/s13063-021-05115-w

**Published:** 2021-02-25

**Authors:** Paolo Mendogni, Alessandra Mazzucco, Alessandro Palleschi, Lorenzo Rosso, Ilaria Righi, Rosaria Carrinola, Francesco Damarco, Emilia Privitera, Jacopo Fumagalli, Gianluca Bonitta, Mario Nosotti, Davide Tosi

**Affiliations:** 1 Thoracic Surgery and Lung Transplant Unit, Fondazione IRCCS Ca’ Granda Ospedale Maggiore Policlinico, University of Milan, Via Francesco Sforza 35, Milan, Italy; 2 Department of Anesthesia and Critical Care, Fondazione IRCCS Ca’ Granda Ospedale Maggiore Policlinico, Milan, Italy; 3 Department of Pathophysiology and Transplantation, Università degli Studi di Milano, Milan, Italy

**Keywords:** Lung cancer, Postoperative pain, Pulmonary lobectomy, Uniportal, Thoracic surgery, VATS

## Abstract

**Background:**

Video-assisted thoracoscopic surgery (VATS) lobectomy is currently the recommended approach for treating early-stage non-small cell lung cancer (NSCLC). Different VATS approaches have been proposed so far, and the actual advantages of one technique over the other are still under debate. The aim of our study is to compare postoperative pain and analgesic drug consumption in uniportal VATS and triportal VATS for pulmonary lobectomy in early-stage lung cancer patients.

**Methods:**

This study is a single-center, prospective, two-arm, parallel-group, randomized controlled trial. It is designed to compare uniportal video-assisted thoracic surgery (u-VATS) and three-port video-assisted thoracic surgery (t-VATS) in terms of postoperative pain. The trial will enroll 120 patients with a 1:1 randomization. The primary outcome is the assessment of analgesic drug consumption. Secondary outcomes are postoperative pain measurement, evaluation of postoperative pulmonary function, and metabolic recovery after pulmonary lobectomy.

**Discussion:**

The choice of which VATS approach to adopt for treating patients undergoing pulmonary resection mostly depends on the surgeon’s preferences; therefore, it is hard to prove whether one VATS technique is superior to the other. Moreover, postoperative analgesic protocols vary consistently among different centers. To date, only a few studies have evaluated the effects of the most popular VATS techniques. There is no evidence about the difference between multiport VATS and u-VATS in terms of postoperative pain. We hope that the results of our trial will provide valuable information on the outcomes of these different surgical approaches.

**Trial registration:**

ClinicalTrials.gov NCT03240250. Registered on 07 August 2017; retrospectively registered.

**Supplementary Information:**

The online version contains supplementary material available at 10.1186/s13063-021-05115-w.

## Background

Currently, video-assisted thoracoscopic surgery (VATS) lobectomy is the recommended approach for treating early-stage non-small cell lung cancer (NSCLC). In 2013, the American College of Chest Physicians indicated VATS as their preferred technique over open surgery (grade 2C) [1]. It is a current opinion that VATS lobectomy is associated with a decreased risk of adverse events, less pain, and shorter length of stay when compared to open surgery [2]; these assumptions are validated by systematic reviews and meta-analyses, even though most of those trials were not randomized [3, 4]. At present, significant differences among VATS surgical approaches specifically lie in the numbers of employed trocars and in the type of utility incisions; in addition, some unusual techniques have been described, such as the transcervical [5], the subxiphoid uniportal [6], and the microlobectomy [7]. The three-port anterior approach described by Hansen (t-VATS) [8] is currently the most commonly adopted technique [9], because of the optimal access on the hilum, low mortality, and proven oncological validity [10, 11]. In 2011, Gonzales Rivas published the first results on pulmonary lobectomy performed via uniportal VATS (u-VATS) [12]. This technique has progressively gained consensus, initially in Asian countries, thereafter in Europe [13]. The debate on the actual advantages of u-VATS over t-VATS is still open [14]. A decrease in postoperative pain and paraesthesia has been reported in minor procedures [15] while to date, there is no adequate evidence on postoperative pain after pulmonary lobectomy. The only randomized study, comparing u-VATS with the “multiports” VATS lobectomy, did not reveal statistically significant differences in terms of postoperative pain [16]. An expert consensus statement in 2019 suggested that u-VATS lobectomy might reduce early postoperative pain and analgesic requirements compared with multiport VATS (m-VATS) [17]. The same group of authors conducted a systematic review and meta-analysis: pooled analyses showed significantly reduced pain according to visual analog scale (VAS) scores in u-VATS patients compared to m-VATS patients on postoperative days 1, 3, 7, and 30. Only two studies including 240 patients reported on the analgesic use: u-VATS patients used analgesics for significantly fewer days per month than m-VATS patients did [18]. The aim of our study is to compare the effects of u-VATS and t-VATS for pulmonary lobectomy in early-stage lung cancer patients, in terms of postoperative pain.

## Methods

### Trial design

The study was designed as a single-center, prospective, two-arm, parallel-group, randomized controlled trial.

**Fig. 1:**
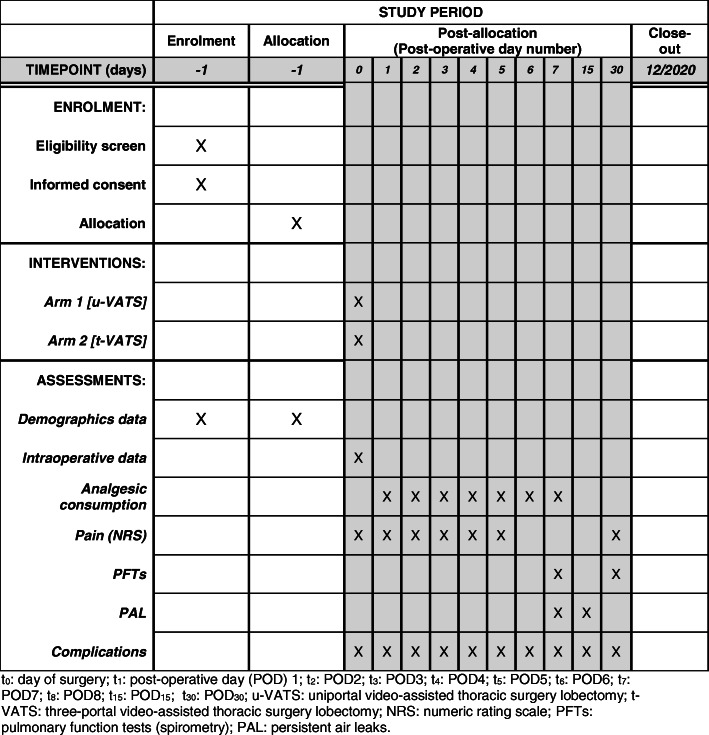
Study process schedule (according to the Standard Protocol Items: Recommendations for Interventional Trials (SPIRIT) guidelines). *t*_*0*_ day of surgery, *t*_*1*_ postoperative day (POD) 1, *t*_*2*_ POD 2, *t*_*3*_ POD 3, *t*_*4*_ POD 4, *t*_*5*_ POD 5, *t*_*6*_ POD 6, *t*_*7*_ POD 7, *t*_*8*_ POD 8, *t*_*15*_ POD 15, *t*_*30*_ POD 30, *u-VATS* uniportal video-assisted thoracic surgery lobectomy, *t-VATS* three-portal video-assisted thoracic surgery lobectomy, *NRS* numeric rating scale, *PFTs* pulmonary function tests (spirometry), *PAL* persistent air leaks

**Fig. 2:**
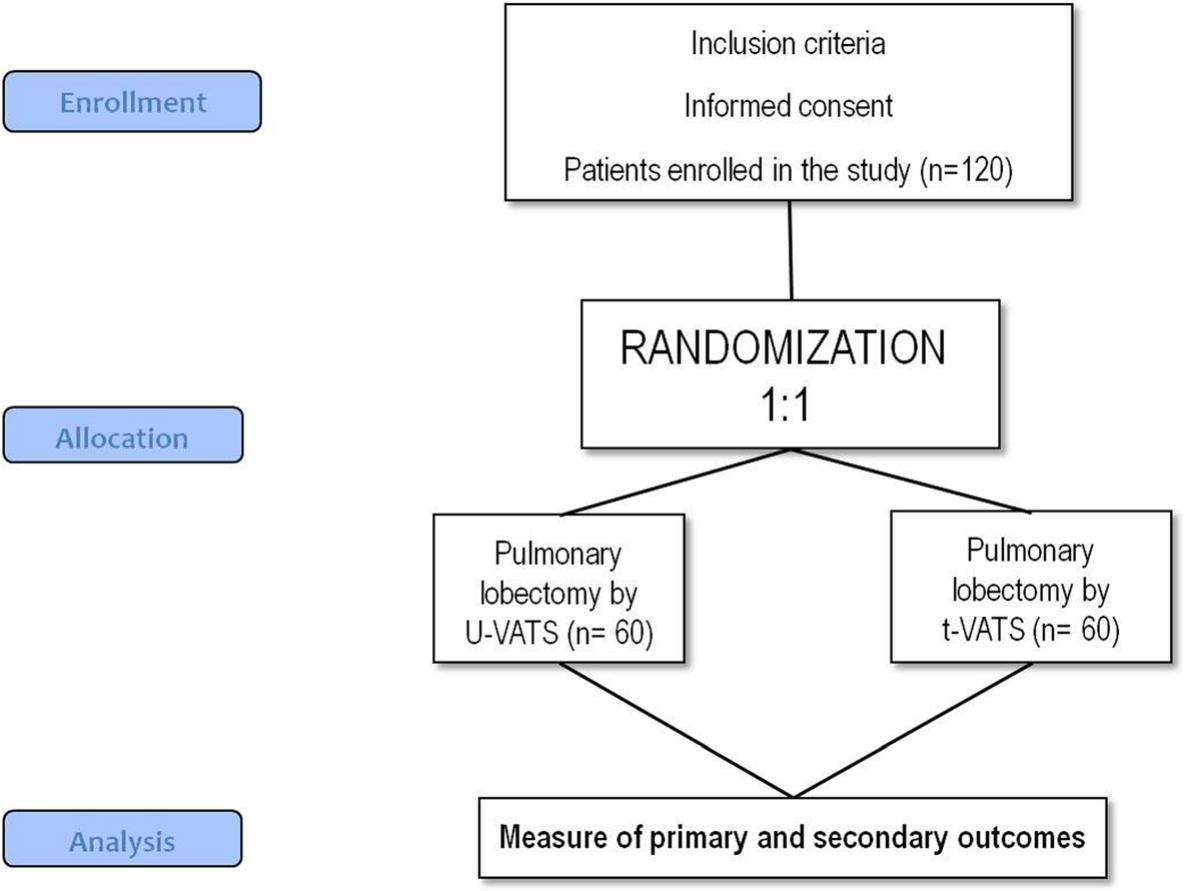
Trial flow chart

The protocol (version 1.0_13/12/2016) is reported in line with the Standard Protocol Items: Recommendations for Interventional Trials (SPIRIT) guidelines following the SPIRIT schedule (Fig. 1), trial flow chart (Fig. 2), and checklist (Additional file 1). The study was registered on ClinicalTrials.gov: NCT03240250. The fundamental hypothesis is that u-VATS could cause lower postoperative pain than t-VATS pulmonary lobectomy for early-stage lung cancer patients. Our hypothesis is based on a simple premise: fewer chest incisions correspond to less pain. This hypothesis is tested out by using analgesic drug consumption as an objective measurement of postoperative pain.

Given an estimated difference of analgesic drug consumption of 20%, the calculated sample size is 120 patients (60 patients each arm), with a 1:1 randomization.

We planned to perform an “interim analysis” once a total number of at least 60 randomized patients, dropouts excluded, is reached. The “interim analysis” will be performed to ensure the safety of the study and to evaluate any significant outcome-related differences between the two techniques that would make the study unnecessary. The surgical staff routinely analyzes the complication rates of every surgical procedure, in a dedicated monthly meeting. From this analysis, if a higher complication rate will be detected in one arm over the other, a data and safety monitoring board (DSMB) will be set up. Furthermore, to protect patients’ safety, the DSMB could be set up during the study, if necessary. All items from the WHO clinical trial registry can be found within the protocol.

#### Eligibility criteria

All consecutive patients referred to our center, the Thoracic Surgery Unit of the University Hospital Fondazione IRCCS Ca’ Granda Ospedale Maggiore Policlinico of Milan (Italy), will be screened by one of two designed members of our surgical staff, according to inclusion and exclusion criteria. Before enrolment, each patient will receive detailed information regarding the study protocol. A specific informed consent regarding the participation to the trial, the randomization, and the surgical incisions will be obtained by a staff surgeon from each participant, after an adequate explanation of the benefits and risks of the study.

The study was approved by the Ethics Committee of Fondazione IRCCS Ca’ Granda Ospedale Maggiore Policlinico, Milan, Italy, on 21 February 2017 (reference number 86_2017), before the trial registration on ClinicalTrials.gov.

The study will be carried out in accordance with the Helsinki Declaration.

#### Inclusion criteria

- Age between 30 and 80 years
- Both genders
- Surgically resectable (R0) non-small cell lung cancer (NSCLC), with cytological or histological diagnosis or clinically suspected, with a diameter lower than 5 cm
- Clinical stage I and II NSCLC
- Patient with American Society of Anaesthesiologists (ASA) score I, II, or III
- Written informed consent (ICF) signed by the patient

#### Exclusion criteria

- Inability to comply with the study procedure
- N2 or N3 clinical nodal involvement of disease
- Induction chemotherapy before surgery
- Chest wall involvement
- Previous thoracic surgery (any type)
- High-grade pleural adhesions
- Renal, liver, or cardiac failure
- Severe chronic obstructive pulmonary disease (COPD), bronchial asthma, or interstitial lung disease (ILD)
- Coagulation disorders
- Allergy to analgesic drugs included in the study protocol
- Chronic use of analgesics, opioids, or steroids
- Wedge resection, sleeve lobectomy, or pneumonectomy
- Re-intervention after VATS lobectomy

#### Outcome definitions and measures

##### Primary outcome

- Postoperative pain after u-VATS and t-VATS, measured by means of total analgesic consumptions, normalized to morphine milligrams, recorded within the 7 days following the operation

##### Secondary outcomes

- Postoperative pain measured by a numeric rating scale (NRS) after 2, 6, 12, and 24 h and on postoperative days (POD) 2, 3, 4, 5, and 30. A pain score will be assigned to each patient after the total amount of NRS is determined. A mean pain score will be calculated and compared between the two groups.
- Evaluation of respiratory function, measured by pulmonary function tests (PFTs) as basal spirometry on POD 7 and POD 30, compared with preoperative measures. Forced vital capacity (FVC) and forced expiratory volume at 1 s (FEV1) will be considered in liters and percentage as measured by PFTs. Preoperative and postoperative measurements will be weighted and normalized for the number of lung segments removed during each lobectomy. The mean differences between pre- and postoperative values will be calculated and compared between the two groups.
- Evaluation of functional and metabolic recovery, measured by metabolic Holter, a multidetection tool recording patients’ physiological data and energy consumption. We will measure physiological data at rest (heart and respiratory rate, arterial blood pressure) and the level of physical activity of the patient, expressed in metabolic equivalents (METs). Means of METs will be calculated and compared between the two groups.
- Intraoperative parameters, calculated by means and percentage:
  - Intraoperative time: measurement of time in minutes from skin incision to the closure of the chest (skin to skin)
  - Intraoperative bleeding: measurement of the total amount of bleeding during the operation expressed in milliliters
  - Total number of resected lymph nodes
  - Conversion rate from VATS to open thoracotomy (total number and percentage)
- Postoperative parameters, calculated by means and percentage:
  - Postoperative air leaks (days of air leaks)
  - Chest drain dwelling time (days)
  - Infection of the surgical site (total number and percentage)
  - Pulmonary complications, including pneumonia, pulmonary embolism, atelectasis (total number and percentage)

#### Randomization

An individual simple sequence randomization with 1:1 allocation will be generated by a person not involved in the enrolment, using the *random allocation rule* [19] by RandomizeR R software package; the consequent assignment to either of the two arms will be done using closed envelopes. The envelopes will be numerated consecutively, containing a sheet marked with an “U” in the case of the u-VATS arm or with a “T” in the case of the t-VATS arm. The allocation of the patient in one or the other arm will be performed in the operating theater before surgery. The envelope assigned to the patient will be opened by the surgeon during the induction of general anesthesia before surgery. If the patient is be assigned to the u-VATS arm, a uniportal VATS lobectomy will be performed. In case of the assignment to the t-VATS group, the surgeon will perform a three-port VATS lobectomy. Given the type of study, patients and investigators cannot be blinded.

#### Description of surgical procedures

Both surgical procedures, u-VATS and t-VATS, will be performed following the same anesthesiologic plan. General anesthesia will be administered, and a double-lumen endobronchial tube will be used to allow single-lung ventilation. Patients will be monitored by standard electrocardiogram (ECG), invasive arterial blood pressure, pulse oximeter, urine catheterization, and, if necessary, central venous catheterization. Patients will be positioned in lateral decubitus, contralaterally to the side of surgery and the bed will be flexed to get wide intercostal spaces.

- *u-VATS:* a single, lateral, muscle-sparing, 4- to5-cm minithoracotomy will be performed through the 4th or 5th intercostal space. No other intercostal incision will be made. All the instruments and camera will be inserted through the minithoracotomy. After pulmonary lobectomy and lymphadenectomy, one intercostal drainage will be inserted through the minithoracotomy [12].
- *t*-VATS: a lateral, muscle-sparing, 4- to 5-cm minithoracotomy will be performed through the 4th or 5th intercostal space. Two additional thoracoscopy ports will be performed in the 7th or 8th intercostal space. The camera and the instrument will be inserted into the pleural space through the minithoracotomy or the thoracoscopy port according to the surgeon’s preference. After pulmonary lobectomy and lymphadenectomy, one intercostal drainage will be inserted through the anterior intercostal incision [8].

#### Description of pre-, intra-, and postoperative anesthesiologic management

Preoperative assessment was performed according to the European Society of Anesthesiology guidelines for the evaluation of candidates to non-cardiac surgery [20].

After routine monitoring as per the American Society of Anesthesiologists guidelines and peripheral venous cannulation (20–16 Ch PVC catheter), induction of general anesthesia with fentanyl (1–2 mcg/kg), midazolam (1–2 mg), and propofol (1–3 mg/kg), and muscle paralysis with rocuronium (0.6–1 mg/kg) is achieved. Patients are intubated with an appropriately sized left-sided double-lumen endotracheal tube under bronchoscopic guidance. General anesthesia is maintained with sevoflurane (end-tidal concentration 1.0–2.0%) to keep the Bispectral Index value between 40 and 60 during the whole surgical procedure, muscle paralysis with sequential rocuronium boluses (0.15 mg/kg), while analgesia is achieved by titrating remifentanil infusion between 0.05 and 0.15 mcg/kg/min according to autonomical responses to surgical stress. Whenever potential risk factors for malignant hyperthermia are identified, total intravenous anesthesia with propofol (4–6 mg/kg/h) is induced. Patients’ invasive arterial pressure is monitored with radial artery cannulation (3 Fr, 5-cm catheter). Central venous cannulation is performed only if central venous pressure monitoring is required. A body surface heating device is applied, and pharyngeal temperature monitoring is established with a target temperature of 35.5–36.5 °C.

Ahead skin incision, together with antibacterial prophylaxis, 30 mg ketorolac and either anti-H2 receptors or proton pump inhibitors are administered. About 1 h before surgery termination, 0.1–0.15 mg/kg, intravenous morphine is administered together with 1 g acetaminophen. For postoperative nausea and vomiting prevention, 4 mg ondansetron is given. Before suturing surgical incisions, an intercostal nerve block is performed by the surgeon on the surgical field by injecting 2–5 mL of 0.375% ropivacaine per intercostal space under thoracoscopic view. Up to 40 mL is administered with a maximum ropivacaine dose of 2 mg/kg. After anesthesia termination, the patient is extubated in the operating room, unless he or she needs protected weaning from mechanical ventilation in the Intensive Care Unit. Immediate postoperative monitoring is performed in the recovery room. If hemodynamic and respiratory function stability is achieved, the patient is transferred to the surgical ward; otherwise, if further monitoring or intensive medical treatment prove necessary, he or she will be transferred to the Intensive Care Unit. Postoperative analgesia is initiated in the recovery room as soon as full recovery of consciousness is observed: intravenous morphine is administered at a rate of 1 mg/h for the first 6 h postoperatively. Six hours after surgery completion and every 4 h thereafter, nurses record the dynamic NRS for pain evaluation. If at any evaluation timepoint the NRS value is below 4, morphine infusion is halved; if NRS value is between 4 and 6, morphine infusion rate is maintained, while, if NRS value is higher than 6, a bolus of 1–2 mg morphine is administered. Parallel administration of intravenous acetaminophen 1 g every 6 h and ketorolac 30 mg every 8 h is performed for the first 3 postoperative days.

#### Postoperative management and follow-up

The patient will be treated during the postoperative course as the standard of care requires, including short-term intravenous antimicrobial prophylaxis, early mobilization, and respiratory physiotherapy. The chest tube will be removed when the total amount of pleural fluid will be < 300 mL/day and no air leaks are detectable.

Patients will be followed up after discharge at POD 7 with clinical examination and PFTs (spirometry) and POD 30 with a clinical examination, NRS pain score, PFTs, and chest X-rays. During the study period, any relevant concomitant treatment will be authorized.

#### Dropout/study termination

Every patient has the opportunity to drop out of the study at any time. The participant’s choice will not compromise their medical treatment that he or she will receive as the standard of care requires. Patients who will drop out will not be displaced to other units. Due to the relatively short follow-up time required (30 days), we expect a small percentage of patients who will be lost at follow-up (about 1–2%), since patients are usually seen 1 month after the discharge. No specific plans are scheduled for participant retention and prevention of loss-to-follow-up. If the percentage of dropout will be < 5%, we will continue the recruitment and consider the study valid.

### Statistical analysis

All the statistical analyses will be performed by blinded analysts. Periodical checking of the input data will be performed in order to verify the completeness and consistency of the dataset. Checks on the consistency and plausibility of the reported data will be carried out before data analysis. The study is powered based on its primary outcome. The sample size was calculated considering a 2-sided *t* test for difference between two independent means, according to the following assumptions: normal distribution for the primary outcome, Choen’s *d* effect size = 0.55 (obtained after a revision of medical reports), power (1-*β*) = 0.85, and *α* = 0.05 [20]. The sample size computation was performed using the R software version 3.2.2 [21]. We have planned to perform an “interim analysis” once a total number of at least 60 randomized patients without dropouts is reached. The “interim analysis” will be performed in order to ensure the safety of the study and to evaluate any significant outcome-related difference between the two techniques that would advise against completing the study. Furthermore, to protect patients’ safety, a data and safety monitoring board (DSMB) could be set up during the study, if necessary.

We will propose two analysis sets: the intention-to-treat set, which would consider all patients randomized regardless of whether they received the randomized treatment, and the “per protocol” analysis set, excluding patients for intraoperative reasons (i.e., massive pleural adhesion, conversion to open surgery). The rationale for a “per protocol” analysis is due to the fact that intraoperative complications have a significant impact on pain which is the primary outcome of our study.

The effect that any missing data might have on results will be assessed via sensitivity analysis of augmented data sets. Dropouts (essentially, participants who withdraw consent for continued follow-up) will be included in the analysis using Bayesian Multiple Imputation by Chained Equations (MICE).

#### Data collection and result dissemination

Patients will be enrolled by a team physician, and their data will be collected in a paper-based case report form (CRF) designed by study staff during pre- and postoperative courses and until 1 month after surgery.

CRFs will be transferred in a password-protected Microsoft Excel® spreadsheet with an ID number assigned to every consecutive enrolled patient. The paper-based CRFs and the electronic database will be kept locked up at the Thoracic Surgery and Lung Transplant Unit of Fondazione IRCCS Ca’ Granda Ospedale Maggiore Policlinico of Milan. The partial and final dataset will be available to the investigators at any time after a request submitted to the Principal Investigator. There are no plans for sharing data from the trial. The trial results will be disseminated in meeting communications and scientific publications. The authorship policies will be shared with the investigators.

#### Risks and benefits of the study

Since both surgical techniques (t-VATS and u-VATS) are used in the current clinical practice, patients will not be exposed to any risk besides those typically entailed in VATS lobectomy. For this reason, there are no additional adverse events that might be expected in the study. We will use the US National Cancer Institute Common Terminology Criteria for Adverse Events 4.1 (CTCAE version 4.1) in order to describe postoperative complications. All the complications will be registered on patient CRFs.

## Discussion

The choice of one VATS approach over the others for patients undergoing pulmonary resection largely depends on the experience of each surgeon; therefore, it is hard to prove whether a technique is more efficient than the other, especially in terms of postoperative pain. Moreover, postoperative analgesic protocols vary considerably among different centers. So far, only a few studies have evaluated the effects of the most popular VATS techniques, the t-VATS and the u-VATS. In 2005, a paper by Jutley et al. retrospectively compared the postoperative pain scores in patients undergoing t-VATS or u-VATS for the treatment of spontaneous pneumothorax, using a visual analog scale (VAS) from 0 to 4. They concluded that postoperative pain and paraesthesia incidence was lower in the u-VATS group, despite the cohort of patients being limited [22].

Recently, we have published an observational, retrospective, multicenter study on data collected in the Italian VATS Group Database, evaluating lobectomies performed for clinical stage I–II NSCLC, by u-VATS and t-VATS: u-VATS lobectomy seemed to entail a higher risk of moderate/severe pain on postoperative days 2 and 3 [9].

The only randomized study comparing u-VATS with the “multiports” VATS lobectomy was a single monocentric trial published by Perna et al. No statistically significant difference between the two groups in terms of postoperative pain was recorded. Nevertheless, one might argue that the sample size was rather under-dimensioned, mainly because the “multiports” group included patients who underwent two different procedures: the Duke two-port technique and the Copenhagen t-VATS approach [16].

The proposed trial will broaden current knowledge of this area by investigating the difference between the t-VATS and the u-VATS in terms of postoperative pain. We have based our hypothesis on a simple premise: u-VATS is less painful than t-VATS because “one incision is less painful than three.” From a strictly logistical point of view, manipulation of the intercostal space is inevitable while employing every VATS technique, as any additional access to the service minithoracotomy can only increase the patient’s amount of pain. Unlike other studies that compared multiport VATS (including two, three, and more ports VATS) to u-VATS, our own study compared two single techniques: in our opinion, this is a methodological advantage, reducing possible confounding bias.

A challenging aspect of any clinical trial is the evaluation of pain suffered by patients. Several clinically tested and validated pain scales have been proposed so far; these try to meet the criteria of objectivity, comprehensibility, and reproducibility. Among the most frequently used pain scales, the Verbal Rating Scale is based on patients’ answers to verbal questions of health care professionals; this scale measures pain intensity only and is subject to discrepancies depending on how each patient understands “mild,” “moderate,” and “severe” pain. Visual analog scale (VAS) is probably the most used pain assessment tool because it is simple to administer, rapid, and easy for patients to understand. However, the VAS likely undervalues pain as a “biopsychosocial” experience and furthermore only measures pain intensity in a specific moment. For our trial, we have decided to use analgesic drug consumption as an objective measure of the “entire pain” suffered by patients during the first postoperative week. By normalizing the amount of the different painkillers used during the first postoperative week to morphine milligrams, we will obtain cumulative morphine consumption, which is a numeric parameter that statistics can properly manage [23].

A secondary endpoint of our study is the evaluation of early postoperative respiratory function, measured by pulmonary function tests. There is no doubt that the greatest impact on respiratory function is attributable to the parenchymal sacrifice following the pulmonary lobectomy itself; nevertheless, the type of surgical insult of the chest wall is significant as well [24]. Hence, multiple chest wall incisions can potentially impact the decreased respiratory function more than a single incision.

In conclusion, the results of this project will broaden current knowledge of the effects of two different VATS approaches to pulmonary lobectomy for lung cancer.

### Trial status

The study started in February 2017. Patients have been enrolled since March 2017 and will be enrolled until September 2020. The study will be interrupted once the sample size of 120 patients enrolled is reached.

## Supplementary Information

**Additional file 1.** SPIRIT Checklist

## Abbreviations

ASA: American Society of Anesthesiologists
COPD: Chronic obstructive pulmonary disease
CRF: Case report form
CTCAE: Common Terminology Criteria for Adverse Events
DSMB: Data and safety monitoring board
ECG: Electrocardiogram
ICF: Informed consent form
ILD: Interstitial lung disease
NRS: Numeric rating scale
NSCLC: Non-small cell lung cancer
METs: Metabolic equivalents
MICE: Multiple Imputation by Chained Equations
m-VATS: Multiport video-assisted thoracic surgery
PFTs: Pulmonary function tests
POD: Postoperative day
PVC: Peripheral venous catheter
SPIRIT: Standard Protocol Items: Recommendations for Interventional Trials
t-VATS: Three-port video-assisted thoracic surgery
u-VATS: Uniport video-assisted thoracic surgery
VAS: Visual analog scale
VATS: Video-assisted thoracic surgery
