# Correction: Uniportal and three-portal video-assisted thoracic surgery pulmonary lobectomy for early-stage lung cancer (UNIT trial): study protocol of a single-center randomized trial

**DOI:** 10.1186/s13063-022-06380-z

**Published:** 2022-07-06

**Authors:** Paolo Mendogni, Alessandra Mazzucco, Alessandro Palleschi, Lorenzo Rosso, Ilaria Righi, Rosaria Carrinola, Francesco Damarco, Emilia Privitera, Jacopo Fumagalli, Gianluca Bonitta, Mario Nosotti, Davide Tosi

**Affiliations:** 1 Thoracic Surgery and Lung Transplant Unit, Fondazione IRCCS Ca’ Granda Ospedale Maggiore Policlinico, University of Milan, Via Francesco Sforza, 35 Milan, Italy; 2 Department of Anesthesia and Critical Care, Fondazione IRCCS Ca’ Granda Ospedale Maggiore Policlinico, Milan, Italy; 3 Department of Pathophysiology and Transplantation, Università degli Studi di Milano, Milan, Italy

**Correction to: Trials 22, 163 (2021)**

**https://doi.org/10.1186/s13063-021-05115-w**

Following the publication of the original article [1], we were notified about a mistake in the analgesia scheme reported in the manuscript.

**Incorrect paragraph on page 5**:

“Postoperative analgesia is initiated in the recovery room as soon as full recovery of consciousness is observed: intravenous morphine is administered at a rate of 1 mg/h for the first 6 h postoperatively. Six hours after surgery completion and every 4 h thereafter, nurses record the dynamic NRS for pain evaluation. If at any evaluation timepoint the NRS value is below 4, morphine infusion is halved; if NRS value is between 4 and 6, morphine infusion rate is maintained, while, if NRS value is higher than 6, a bolus of 1–2 mg morphine is administered. Parallel administration of intravenous acetaminophen 1 g every 6 h and ketorolac 30 mg every 8 h is performed for the first 3 postoperative days.”

**Corrected paragraph**:

“We used NRS for evaluation of pain, with 0 to 10 rates. Pain was rated and registered every four hours after the procedure; mean NRS was then calculated daily from POD 1 to POD 7.

Intravenous infusion of 1 mg/h of morphine started about one hour before the end of surgery. At the end of the procedure, surgeons performed intercostal nerve block of 3-4 intercostal spaces under thoracoscopic vision, using 2-5 mL of ropivacaine 7.5% per intercostal space. After surgery, postoperative analgesia started in the recovery room and consisted of intravenous morphine infusion, which was maintained at 1 mg/h for 6 hours, 1000 mg intravenous acetaminophen three times a day and 30 mg intravenous ketorolac three times a day.

After the first 6 hours, morphine infusion rate was reduced to 0.5 mg/h. The infusion rate was then adjusted according to NRS, which was assessed every 4 hours, as follows:

- NRS <4: infusion rate was decreased by 0.125 mg/h
- NRS =4: infusion rate was not altered
- NRS >4: infusion rate was increased by 0.125 mg/h
- NRS >6: 1 mg bolus of morphine was administered

Morphine infusion was stopped when a NRS lower than 4 was reported by a patient receiving a 0.125 mg/h dose, or if any side effect occurred (dizziness, confusion, vertigo, nausea, and vomiting). After chest drain removal, acetaminophen (1000 mg three times a day) was administered orally, and ketorolac 30 mg was administered only with a NRS > 4.

Non-steroidal anti-inflammatory drugs (NSAIDs) consumption was taken in consideration and converted to morphine equivalents according to equianalgesic charts, thus calculating the cumulative morphine consumption (CMC) [reference now numbered 23 Nosotti et al EJCTS 2015].”

The original article has been corrected.
